# Oasis: online analysis of small RNA deep sequencing data

**DOI:** 10.1093/bioinformatics/btv113

**Published:** 2015-02-19

**Authors:** Vincenzo Capece, Julio C. Garcia Vizcaino, Ramon Vidal, Raza-Ur Rahman, Tonatiuh Pena Centeno, Orr Shomroni, Irantzu Suberviola, Andre Fischer, Stefan Bonn

**Affiliations:** ^1^Laboratory of Computational Systems Biology and ^2^Laboratory of Epigenetic Mechanisms in Dementia, German Center for Neurodegenerative Diseases, 37077 Goettingen, Germany

## Abstract

**Summary:** Oasis is a web application that allows for the fast and flexible online analysis of small-RNA-seq (sRNA-seq) data. It was designed for the end user in the lab, providing an easy-to-use web frontend including video tutorials, demo data and best practice step-by-step guidelines on how to analyze sRNA-seq data. Oasis’ exclusive selling points are a differential expression module that allows for the multivariate analysis of samples, a classification module for robust biomarker detection and an advanced programming interface that supports the batch submission of jobs. Both modules include the analysis of novel miRNAs, miRNA targets and functional analyses including GO and pathway enrichment. Oasis generates downloadable interactive web reports for easy visualization, exploration and analysis of data on a local system. Finally, Oasis’ modular workflow enables for the rapid (re-) analysis of data.

**Availability and implementation:** Oasis is implemented in Python, R, Java, PHP, C++ and JavaScript. It is freely available at http://oasis.dzne.de.

**Contact:** stefan.bonn@dzne.de

**Supplementary information:** Supplementary data are available at *Bioinformatics* online.

## 1 Introduction

Small RNAs play pivotal roles in many biological processes, ranging from organismal development to disease states including cancer. As such, they have gained recent interest not only in basic research, but also as therapeutic targets and biomarkers of disease in clinical settings (Witwer, 2014).

The current method of choice for small RNA analysis is deep sequencing, which allows for the comprehensive charting of small RNAs at a reasonable price. Consequently, it is not the generation of data but the subsequent analysis that is usually limiting. To this end several web applications have been developed that allow for the analysis of small-RNA-seq (sRNA-seq) data. Especially recent additions to the small RNA analysis landscape convince with their user friendliness, analysis portfolio and their performance. These include MAGI (Kim *et al.*, 2014), an all-in-one application featuring structured interactive output, ISRNA (Luo *et al.*, 2014) which combines powerful search functionality with an online project database and CPSS (Zhang *et al.*, 2012) a web application that detects miRNA edits and modifications.

Although many good web platforms for the analysis of sRNA-seq data exist some important analysis features have yet to be integrated. For example, no current web application allows for multivariate data analysis, including multi-group comparisons and the incorporation of covariate and interaction information. Also, there is currently no web application that allows for the identification of biomarkers of disease via integrated machine-learning modules. Finally, current sRNA-seq web services do not allow for automated analysis or batch submission of jobs via an advanced programming interface (API), a feature that would greatly facilitate analysis workflows for frequent users. In the end, these functionalities should be paired with a solid prediction of novel miRNAs, their targets and functional analyses using gene ontology and pathway enrichment.

## 2 Design and Key Features

Oasis addresses all of these restrictions in a user-friendly, modular analysis environment. The standard workflow comprises the compression of FASTQ files on the user’s local system and their upload for subsequent small RNA detection and sample quality assessment (sRNA Detection module). The sRNA Detection module aligns reads to the genome, annotates known small RNA species and predicts novel miRNAs for all the sequences that do not map to annotated small RNAs. The output of the sRNA Detection module generates downloadable, interactive web reports that contain quality plots, detailed information on novel small RNAs, as well as count files containing small RNA read counts for each sample.

These count files can then be uploaded to the differential expression (DE Analysis) or classification modules. Both modules provide downloadable, interactive results in web reports, highlighting important small RNAs, deliver annotations, visualizations and tables for subsequent analysis on a local computer.

The separation of the small RNA detection and quality assessment from the functional analysis of data provides the user with two main advantages. First, the user can have a careful look at sample quality before the functional analysis. Good quality samples can be chosen and uploaded for differential expression or classification and bad quality samples can be dismissed. Although increasing the hands-on-time of the user we deem this step absolutely essential, as single outliers can severely impair the results of any following statistical analyses. Second, due to the small size of the sample count files Oasis allows for the very fast re-analysis of different subsets of samples or between different experiments.

In Table 1, we compare existing web services for sRNA-seq analysis to Oasis. We tried to provide an objective, comprehensive overview of features that we deem essential, important or beneficial, also highlighting areas in which other tools provide better performance than Oasis. Finally, the comparisons in Table 1 are limited to the newer ‘second generation’ web applications that satisfy at least four features we deem relevant. In the following section, we highlight the most salient features of Oasis.

**Table 1.** Comparison of sRNA-seq web applications Oasis, MAGI (Kim *et al.*, 2014), ISRNA (Luo *et al.*, 2014), CPSS (Zhang *et al.*, 2012), CAP-miRSeq (Sun *et al.*, 2014) and mirTools2 (Wu *et al.*, 2013)

Feature	Oasis	MAGI	ISRNA	CPSS	CAP-miRSeq	mirTools2
FASTQ compression	✓	✓	✓	✓		
miRNA modification or SNV detection				✓	✓	
miRNA prediction	✓	✓		✓	✓	✓
Differential expression (multiple samples)						
• Two groups	✓	✓	✓		✓	✓
• Multivariate	✓					
Classification	✓					
Novel miRNA target prediction	✓	✓		✓		✓
Pathway/GO analysis	✓	✓		✓		✓
Interactive visualization						
• Server-side	✓	✓	✓			
• Client-side	✓					
Modular analysis	✓		✓		✓	
Integrated browser			✓			
Batch job submission (API)	✓					
Project database			✓			

### 2.1 Data compression and server upload

Oasis features a standalone and platform-independent application that allows for the compression of FASTQ files prior to their upload to the server. OasisCompressor is written in Java and C++ and takes two arguments, the input files and the output location. An additional option is the number of parallel processes that OasisCompressor will execute. The compression ratio of FASTQ files depends on the entropy of samples but usually ranges between 200- and 800-fold. Once compressed samples can be rapidly uploaded from the client to the server using Oasis’ web frontend. The technical details of OasisCompressor can be found in the Supplementary material.

### 2.2 Interactive web reports

The results of all Oasis analysis modules are provided as downloadable, interactive web reports. These JavaScript-empowered web reports can be opened in the users local web browser and support flexible visualization and the interactive analysis of results. For example, the HTML report containing differentially expressed small RNAs can be interactively sorted, subset manually or by *P* value and miRNA targets can be further analyzed for the functional enrichment of categories. The programs for the functional enrichment can also be interactively chosen, giving the user the ability to compute and visualize enrichment for GO and KEGG using G:Profiler (Reimand *et al.*, 2011) or DAVID (Huang *et al.*, 2007), protein–protein interaction using GeneMANIA (Zuberi *et al.*, 2013), STRING (Franceschini *et al.*, 2013) and STITCH (Kuhn *et al.*, 2014) for varying *P* values and small RNA lists, all in the local browser.

### 2.3 Multivariate differential expression

Oasis supports multivariate differential expression analysis of samples as implemented in the DESeq2 (Love *et al.*, 2014) package. This includes multi-group comparisons and the incorporation of covariate and interaction information. Thus, questions about the interaction of two or more factors can be asked, or the influence of several covariates can be included in an analysis. A simple question could be to examine the effect of a disease on small RNA expression, while correcting for variations in age or medication (covariates).

### 2.4 Classification

Another unique feature of Oasis is the detection of biomarkers using classification routines. The involvement of small RNAs in disease processes such as cancer has sparked considerable interest in the use of small RNAs as therapeutic target or biomarker (Witwer, 2014). In Oasis, the user can easily detect small RNA biomarkers using a Random Forest machine learner (Breiman, 2001). Random Forests are inherently robust classifiers that have only two parameters of importance and are extremely stable over parameter space, providing a simple yet powerful classification routine for the non-technical user. As input, the classification routine takes the count files of the sRNA Detection module, which again allows for a rapid and flexible (re-) analysis of samples due to the small size of the count files.

### 2.5 Automated job submission

A prevalent bottleneck of sRNA-seq analyses on web servers is that users are forced to manually upload samples and submit jobs. Oasis supports the automated submission of jobs via an API. By using simple python scripts frequent users can automate analysis workflows for every Oasis module, including the compression of FASTQ files prior to data upload.

Finally, we compared the runtimes of Oasis and MAGI using three different published sRNA-seq datasets and found that Oasis performs favorable in all three instances (see Supplementary material). Comparison of Oasis’ analysis results to published data shows that Oasis detects 85% (11/13) of the differentially expressed sRNAs that have been biologically validated (see Supplementary material and Oasis’ demo page).

In summary, Oasis is a fast and flexible web application for sRNA-seq data analysis that supports multivariate DE analysis and classification. It allows for easy automation of jobs via an API, provides aid to new users via tutorials and demo analyses on published datasets and allows the user to interactively analyze results on his local computer. As such, Oasis should be a valuable addition to the landscape of sRNA-seq analysis web applications.

## Supplementary Material

Supplementary Data
