# Supplementary material for: Oasis: online analysis of small RNA deep sequencing data
Source: Bioinformatics. 2015 Feb 19;31(13):2205–7. doi: 10.1093/bioinformatics/btv113 (PMC4481843; doi:10.1093/bioinformatics/btv113)
Supplement: Supplementary Data [file supp_31_13_2205__index.html]

Oasis: online analysis of small RNA deep sequencing data — Oasis: online analysis of small RNA deep sequencing data — Supplementary Data 

# Oasis: online analysis of small RNA deep sequencing data

## Supplementary Data

files

**Files in this Data Supplement:**

- Supplementary Data - docx file
