# Supplementary material for: Oasis: online analysis of small RNA deep sequencing data
Source: Bioinformatics. 2015 Feb 19;31(13):2205–7. doi: 10.1093/bioinformatics/btv113 (PMC4481843; doi:10.1093/bioinformatics/btv113)
Supplement: Supplementary Data [file supp_btv113_OasisRevision_SupplementaryMaterialFinal_26012015_clean_copy.docx]

# Supplementary information

## Platform

Oasis was developed with the end-user in mind, trying to implement hardware and software that allows for an accurate yet very fast analysis of data. Therefore, Oasis is embedded into a high-end hardware infrastructure using state-of-the-art analysis routines. A detailed overview of the hardware architecture is supplied in Supplementary Figure 1.


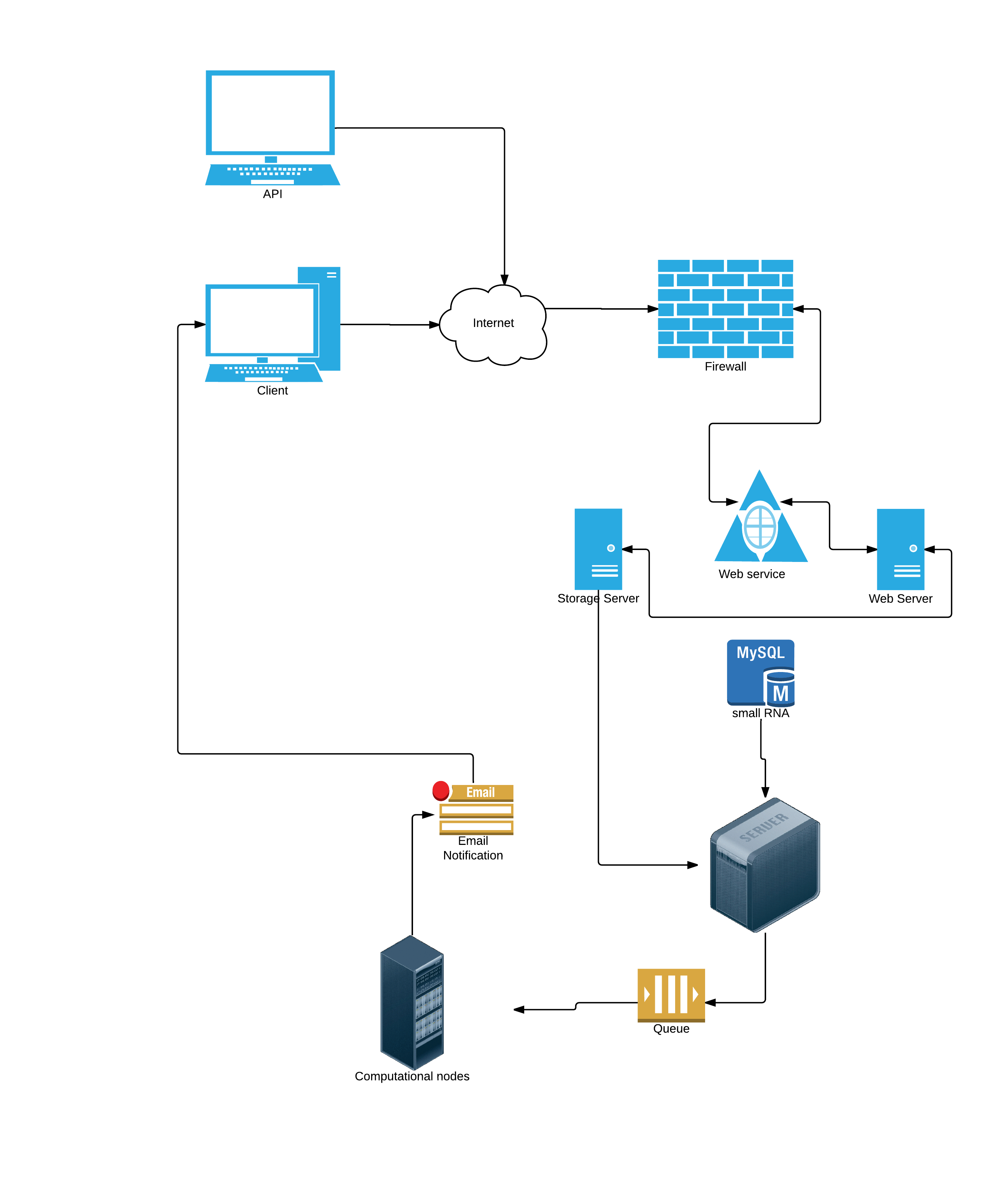


Supplementary Figure 1. Oasis hardware architecture

Files compressed by OasisCompressor are uploaded directly to the Oasis server. The server analyzes the data and sends back a link of the results to the user’s e-mail inbox. In brief, the web server processes the uploaded content and launches a job in the computational node. The output is transferred to a storage server and this server communicates with the web server and the computational nodes. For security reasons the computational node starts the analysis without communicating with the web server. A storage server saves the uploaded data and a job file that is read afterwards by the computational server. This job file contains all the parameters to launch the correct analysis workflow: e.g. the output directory, adaptor, reference genome and e-mail supplied by the user.

**Analysis modules**

Oasis is split into three analysis modules that allow for the flexible and rapid analysis of samples. This is especially helpful when users want to analyze different combinations of samples or using different analysis parameters.

***sRNA detection***

The ‘sRNA Detection’ module reads FASTQ files (either compressed or uncompressed), maps reads to the genome, annotates known sRNAs, and predicts novel miRNAs. It returns various quality metrics, detailed miRNA prediction results, and sRNA count files in a downloadable, interactive web report. This module supplies the user with the necessary information to decide if the quality of samples is sufficient to work with or if some samples should be removed. The count files that are produced by the ‘sRNA Detection’ module are used as input to the ‘DE Analysis’ or ‘Classification’ modules. Due to the small size of the count files this allows for very fast (re)-analysis cycles and seamless data upload. Supplementary table 1 contains an overview of the external software implemented into the sRNA Detection module.

**Supplementary Table 1.** Software used by the ‘sRNA Detection’ module.

| Task | Software | Version |
| --- | --- | --- |
| Adapter trimming | Cutadapt (Martin, 2011) | 1.2.1 |
| Alignment | STAR (Dobin *et al.*, 2013) | 2.30.0e_r291 |
| miRNA prediction | mirDeep2 (Friedländer *et al.*, 2012) | 2.0.0.5 |
| Quality assessment | FastQC (Andrews) | 0.11.2 |
| Counting | featureCounts (Liao *et al.*, 2014) | 1.4.6 |

***Differential expression***

The ‘DE Analysis’ module takes as input the count files that are output from the ‘sRNA Detection’ module. It returns various quality metrics, detailed differential expression results, and known and predicted targets of miRNA in a downloadable, interactive web report. This web report allows for the subsequent functional analysis of the differentially expressed miRNAs by using JavaScript enabled API queries to GeneMania (interactome and GO analysis) (Zuberi *et al.*, 2013), g:Profiler (GO, pathway-Kegg, Reactome, etc) (Reimand *et al.*, 2011), STRING (protein-protein interaction network) (Franceschini *et al.*, 2013), STITCH (chemical-protein interaction network) (Kuhn *et al.*, 2014), and DAVID (enrichment analysis based on many biological databases) (Huang *et al.*, 2007).

The differential expression analysis powered by DESeq2 (Love *et al.*, 2014) (Supplementary Table 2) allows for the analysis of statistically significant expression between multiple groups containing multiple samples. Some of the more advanced comparisons that can be performed using the ‘DE Analysis’ module are:

1. Paired and unpaired analyses (e.g. time-series data)
2. Multiple group comparisons (e.g. control vs. treatment 1 vs. treatment 2)
3. Two or multiple groups including covariates (e.g. control vs. disease including co-variates such as age, gender, and medication).

Whereas the analysis of two or multiple groups is automatically handled by just uploading two or more groups of samples, the covariate analysis requires some more effort and knowledge of the user. Use cases on how to perform multivariate analyses are included in the demo datasets, supplying step-by-step guides through the analysis workflow.

**Supplementary Table 2.** Software used by the ‘DE Analysis’ module.

| Task | Software | Version |
| --- | --- | --- |
| Differential expression | DESeq2 (Love *et al.*, 2014) | 1.4.5 |
| Target prediction | miRanda (Betel *et al.*, 2008) | 3.3a |
| Functional analysis | GeneMania (Zuberi *et al.*, 2013)  G:Profiler (Reimand *et al.*, 2011)  STRING (Franceschini *et al.*, 2013)  STITCH (Kuhn *et al.*, 2014)  DAVID (Huang *et al.*, 2007) | -  -  -  -  - |

***Classification***

The ‘Classification’ module takes as input the count files that are generated by the 'sRNA Detection' module and performs a binary classification using Random Forests (Breiman, 2001). Upon upload of count files the ‘Classification’ module first performs an across-sample normalization of the data. The normalization method is part of the DESeq2 package and is based on the hypothesis that most genes are not differentially expressed. In brief, a scaling factor for a given sample is computed as the median of the ratio, for each small RNA, of its read count over its geometric mean across all samples. The underlying idea is that non-DE genes should have similar read counts across samples, leading to a ratio of 1. Assuming most genes are not DE, the median of this ratio for the sample provides an estimate of the correction factor that should be applied to all read counts of this lane to fulfill the hypothesis. By calling the estimateSizeFactors() function in DESeq2, this factor is computed for each lane, and raw read counts are divided by the factor associated with their sequencing lane. As a warning, this normalization does not apply to situations where most small RNAs are changing, as for example in the case of a Dicer knockdown experiment.

Upon normalization, small RNAs that do not have at least 1 read per replicate in one of the groups are discarded from further analysis (e.g. 5 biological replicates for two groups should have at least 5 reads in one of the groups for any small RNA).

Following the preprocessing of the input data, Oasis determines the set of most discriminative small RNAs using a Random Forest classifier as implemented in the R randomForest package. The ‘Classification’ module can take two parameters, the number of features per tree mtry (defaults to the square-root of the number of features m) and the number of trees per forest ntree (defaults to 100.000 trees). Although the user can change these values in Oasis, we don’t recommend changing them without knowledge of the effects that this might imply. Especially high mtry values might result in over-fitting of the model.

The ‘Classification’ module reports performance measures associated to the discriminating power of the classifier in a downloadable, interactive web report. The output includes ROC, precision and recall, variable importance plots, a PCA and an outlier plot (based on the associated score provided by Random Forest). Additionally, it provides an error rate for the number of trees that allows the user to estimate if enough trees have been used for the classification. Moreover, this module reports the cross-validated prediction error of random forest models trained by increasingly adding features (small RNAs). This report comes in the form of a 'Feature selection' plot that is useful for the identification of an 'optimal' set of RNAs for classification (‘biomarker’ signature).

As in the DE module, the web report allows for the subsequent functional analysis of sRNA targets based on their feature importance. Version details of the software used in this module are described in Supplementary Table 3.

**Supplementary Table 3.** Software used by the ‘Classification’ module.

| Task | Software | Version |
| --- | --- | --- |
| Normalization | DESeq2 (Love *et al.*, 2014) | 1.4.5 |
| Classification | randomForest (Liaw and Matthew, 2002) | 4.6-10 |
| Visualization | ROCR (Sing *et al.*, 2005) | 1.0-5 |

## Alignment, counting, and annotation of reads

Prior to alignment, reads are filtered for a minimum length of 15 nucleotides and a maximum length of 32 nucleotides. The remaining preprocessed reads are subsequently aligned to a reference genome using STAR (Dobin *et al.*, 2013) in non splice-junction-aware mode. Reads of length 15 - 19 nucleotides are aligned allowing for 0 mismatches whereas reads of length 20 – 32 nucleotides are mapped allowing for 1 mismatch. Reads that could only be aligned using soft trimming (trimming of the beginning or end of reads that is not counted as mismatches) were removed from STAR’s output using a custom script.

The STAR options used are:

--outFilterMismatchNoverLmax 0.05 (0 mismatches for reads with length 15-19, 1 mismatch for reads with length 20-32)

--outFilterMatchNmin 15

--outFilterScoreMinOverLread 0

--outFilterMatchNminOverLread 0

--alignIntronMax 1

Currently, Oasis supports the following model organisms and genome versions:

- mm10 for *Mus musculus*
- hg19 for *Homo sapiens*
- dm74 for *Drosophila melanogaster*
- zv9 for *Danio rerio*
- ce10 for *Caenorhabditis elegans*

For the counting step Oasis assumes stranded sRNA-seq data (which is currently the case for most sRNA library preparations) and uses the following databases and versions:

- Mirbase for miRNA sequences (version 20, release date: 2013-10-1)
- Ensembl for snRNA, snoRNA and rRNA:

*Homo sapiens* version GRCh38.74

*Mus musculus* version GRCh38.74

*Drosophila melanogaster* version BDGP5.77

*Danio rerio* version Zv9.78

*Caenorhabditis elegans* version WBcel235.78

- piRNAbank V.2 for piRNAs

Databases and versions will be updated periodically and the user will be able to choose among the new and old databases in the advanced settings.

## miRNA prediction

The prediction of novel miRNAs is performed by miRDeep2 (Friedländer *et al.*, 2012) software on the combined set of all input FASTQ files (i.e., all the input samples are merged into a unique file containing the total reads). miRDeep2 runs with the default parameters except for the minimum read depth (-a). This parameter is automatically computed according to the total number of uniquely mapped reads, targeting a performance of at least 80% true positives and 80% recall. The results are visualized in a miRDeep2 web report containing detailed information about each predicted miRNA (e.g. prediction scores, total mapped reads, and the miRNA family based on seed similarity). Each predicted structure can be viewed by clicking in the "provisional id" link. The filters are based on significant randfold p-value (default mirdeep2 threshold) and rFAM alerts for other types of RNA (e.g. rRNAs and tRNAs). As Oasis maps to the mature miRNA sequence, miRDeep2 code was modified in order to show the mature miRNA position instead of the pre-mature miRNA position.

## miRNA target annotation and prediction

Oasis annotates validated and predicted miRNA targets for all miRNAs. Known miRNAs are annotated with experimentally validated target RNAs using MirTarBase (Hsu *et al.*, 2014) and miRecords (Xiao *et al.*, 2009) (version 4.5 and version 4 respectively). Additionally, known miRNAs are annotated with predicted miRNA targets using the miRanda database (Betel *et al.*, 2008). Oasis supplies only high stringency predicted miRNA targets from miRanda by filtering for targets having "Good mirSVR score, Conserved miRNA" and "Good mirSVR score, Non-conserved miRNA" and a mirsvr_score lower than -1.2.

mRNA targets for novel miRNAs are predicted by miRanda 3.3a using the mature miRNA sequence and the complete set of transcript UTRs for a given organism. In order to minimize the number of false positives present in miRNA target predictions, Oasis uses only conserved sites of transcript UTRs obtained in TargetScan (release 6.2) alignment (Friedman *et al.*, 2009; Witkos *et al.*, 2011). Only targets with alignment scores higher than 155 and energy scores lower than -20 are considered. The list of predicted targets is sorted and displayed in decreasing score order.

## Interactive web reports and functional analysis

The results of every Oasis analysis module can be downloaded as interactive web reports. This includes interactive tables of e.g. differentially expressed sRNAs. In brief, Oasis’ interactive tables allow for the sorting of columns by a field of interest, e.g. by adjusted p-value. Other options are the sub-setting of the DE results for an adjusted p-value of interest (0.1, 0.05, 0.01 or 0.001). Alternatively, sRNAs can be selected manually. Once selected, the user can perform functional enrichment analyses based on validated and/or predicted miRNA target RNAs. Currently, Oasis supports the following functional analyses: Genemania (interactome and GO analysis) (Zuberi *et al.*, 2013), G:Profiler (GO, pathway-Kegg, Reactome, etc) (Reimand *et al.*, 2011), STRING (protein-protein interaction network) (Franceschini *et al.*, 2013), STITCH (chemical-protein interaction network) (Kuhn *et al.*, 2014), and DAVID (enrichment analysis in multiple databases) (Huang *et al.*, 2007). We provide theses external services by using their respective APIs with a maximum 1000 targets. In case the user wants to use a different web application for functional analyses or submit a bigger number of targets, Oasis allows for the easy display of the selected gene targets.

## Data policy

Upon the completion of an analysis the user receives a mail including a link to the analysis results that allows the user to download the results in zipped format, the file will be on Oasis server up to two weeks after job completion. There is currently no restriction on the number of samples or jobs per user.

## FASTQ compression

The analysis of sRNA-seq data requires the upload of big datasets to the Oasis server. Depending on the size of this data, this process can take from minutes to several hours, a process that can easily fail due to network connection problems.

In order minimize the time needed for data upload to the server, Oasis provides a Java executable that extracts quality metrics and compresses the read information from FASTQ files, ‘OasisCompressor’. The compression rates vary depending on the sample entropy and sequencing depth. For example, OasisCompressor reduces the size of 70 Alzheimer Disease FASTQ files (Leidinger *et al.*, 2013) from 287 to 0.42 GB, achieving almost 700 fold compression of the data.

‘OasisCompressor’ was developed for the end user as an easy-to-use, parameter-free, platform-independent Java program featuring a simple graphical user interface (GUI). The application takes three arguments: Input files, output location, and number of cores used for parallel file compression. Users can choose uncompressed input files in *.fq, *.fastq for very fast compression but can also supply *.fq.gz, *.fastq.gz, *.fq.zip, *.fastq.zip format. These file types can either be chosen directly or the user can select a directory containing files in these formats. The output of ‘OasisCompressor’ can be uploaded to the server using the web interface or an API.

***FASTQ compression details***

The actual compression routines are coded in C++ for optimal speed and make use of the Succinct Data Structure Library (SDSL, <https://github.com/simongog/sdsl-lite>) and the Boost (<http://www.boost.org/>) libraries.

Sequences in small RNA are short and repetitive (entropy depends on the data). Therefore an obvious and naïve idea is to store the frequencies of every sequence. By doing that we can reduce the file size and keep the same sequence information. To save all these sequences we use a hash table, where the key is the sequence and the value is the frequency (count) of that sequence in the file. We further improved compression by encoding the nucleotides in a 3-bits format, A=000, C=001, G=010 and T=011, extended nucleotide codes are coded as 100. Moreover, the hash table is translated (which has an overhead by using pointers and other artifacts) into two vectors: one with the sequences (keys) and one with the frequencies (values). It is important to point out that these two vectors are compressed vectors: the sequences vector is a sparse bit vector and the frequencies vector is a compressed integer vector.

***Sparse bit vector***

This sparse bit vector uses the Elias-Fano encoding (Fano, 1971). The Elias-Fano encoding is a high bits / low bits representation of a monotonically increasing sequence of $numValues>0 natural numbers x[i]$.

$$0\leq x\left[ 0 \right]\leq x\left[ 1 \right]\leq\ldots\leq x[numValues-2]\leq x[numValues-1]\leq upperBound$$

Where $upperBound>0$ is an upper bound on the last value. The Elias-Fano encoding uses less than half a bit per encoded number more than the smallest representation that can encode any monotone sequence with the same bounds. The lower $L$ bits of each $x[i]$ are stored explicitly and contiguously in the lower-bits array, with $L$ chosen as$\left( {log}_{2}\left( \right) \right):$

$$L=max(0, floor(\log\left( \frac{upperBound}{numValues} \right)))$$

The upper bits are stored in the upper-bits array as a sequence of unary-coded gaps

$$\left( x\left[ -1 \right]=0 \right):(\frac{x\left[ i \right]}{2^{L}}-\frac{x\left[ i-1 \right]}{2^{L}})$$

The unary code encodes a $natural number n by n 0$n bits followed by a 1 bit: 0...01. In the upper bits the total the number of 1 bits is $numValues$ and the total number of 0 bits is:

$$floor(\frac{x\left[ numValues-1 \right]}{2^{L}})\leq\frac{upperBound}{2^{max(0,floor\left( \log\left( \frac{upperBound}{numValues} \right) \right))}}\leq2^{numValues}$$

The Elias-Fano encoding uses at most $2+ceil(log(\frac{upperBound}{numValues}$)) bits per encoded number. With the $upperBound$

$$\left( p is an integer \right):2^{p}<x\left[ numValues-1 \right]\leq upperBound\leq2^{p+1}$$

the number of bits per encoded number is minimized. This information is then serialized and saved in a binary format with the extension ‘.dat’.

***Compressed integer vector***

Since OasisCompressor uses a hash table to compress the sequences, the frequency of every sequence is saved as an integer vector with variable length codes. This vector aims to save a generic immutable list of integers. Vectors support storing integers with the minimum amount of bits required representing the maximum number in the vector. The bit-width of integers can be adjusted at runtime for all vectors of class int_vector<> (=int_vector<0>) and int_vector<X> with X not in {1,8,16,32,64}. These vector types are called bitcompressed. For X in {8,16,32,64} int_vector<X> is specialized and corresponds to an array of integers of type uint8_t, uint16_t, uint32_t, and uint64_t. The read and write access of those non-bitcompressed vectors is faster compared to the bitcompressed versions. The case X=1 corresponds to a bit vector (int_vector<1> is typedef to bit_vector) that accelerates the read and write operation compared to a bitcompressed version. This information is then serialized and saved in a binary format with the extension .count.

***Quality score statistics***

The aim of the compression routine was to supply all quality information to FastQC that is necessary. This allowed to pre-compute statistical information on the localhost and ignore the per base quality information for the final data compression as this information is not used by FastQC. The following quality information is extracted and used in a customized version of the widely used FastQC program on the server side:

- Mean and median quality score for every position in the sequence.
- Frequency of quality scores in the whole FASTQ file.
- Quantile percentiles of quality score for every position in the sequence length: Lower quartile (0.25), upper quartile (0.75), 10^th^ quartile (0.1), and 90^th^ quartile (0.9).

This information is saved in a text format with the extension .stats.

## Batch job submission (API)

Oasis API is written in Python and it has been tested with Python 3. It contains 3 main scripts: smallRNA.py (mapping, predict novel miRNA and quality control), smallRNAde.py (differential expression analysis) and smallRNAclassification.py (classification analysis). They can be downloaded in the main webpage of Oasis.

These scripts send the data to the server for one of the three specific analyses. On the server-side there are three small web services to accept connections, one for each type of analysis. These web services only accept HTTP post method in multipart/form-data.

***smallRNA.py***

This script sends compressed sRNA FASTQ files to the server and supplies the parameters needed for the ‘sRNA Detection’ analysis.

In brief, after checking for the command line arguments, the script assures that the web-application is available and in case of communication problems reports an error and exits. Subsequently the files are tested for compliance and an HTTP post request sending a multipart-encoded file is dispatched. In case the user submits several samples with the same settings the process is repeated for every file.

***smallRNAde.py***

This script sends count files to the server and supplies the parameters needed for the ‘DE Analysis’ module. Analogous to ‘smallRNA.py’, the script tests for a valid connection and file validity and sends an HTTP post request to the server. The script supports basic two group analyses as well as multivariate analyses, supporting the full functionality of the ‘DE Analysis’ module.

***smallRNAclassification.py***

This script sends count files to the server and supplies the parameters needed for the ‘Classification’ module. Analogous to ‘smallRNAde.py’, the script tests for a valid connection and file validity and sends an HTTP post request to the server.

## Validation on published data

In order to assess the biological validity of Oasis’ results, we tested and compared Oasis on three published sRNA-seq datasets, two of which contain independent validation results for select miRNAs.

In brief, we validated Oasis’ results using a published dataset of 70 Alzheimer Disease (AD) and control samples (Leidinger *et al.*, 2013), 20 Psoriasis and control samples (Joyce *et al.*, 2011), and 22 Renal Cancer and control samples (Osanto *et al.*, 2012). All of these studies used different statistical approaches and older versions of mirbase. Still, Oasis reached reasonable overlap for the statistically significant sRNAs (Supplementary Table 4, Overlap) and was able to detect 85% (11/13) of independently validated miRNAs. In summary, Oasis produces biologically meaningful results, complying well with published, validated, differentially expressed miRNAs. Of note, none of the above-mentioned publications looked into the differential expression of other small RNA classes (piRNA, snRNA, etc.), so the analyses were restricted to miRNAs.

**Supplementary Table 4.** Overlap of differentially expressed sRNAs using three published datasets.

|  | Statistics^1^ | Overlap^2^ | Validated overlap^3^ |
| --- | --- | --- | --- |
| Alzheimer disease | Wilcoxon-Mann-Whitney | 68% | NA |
| Psoriasis | Pearson’s chi-squared | 62% | 75% (6/8) |
| Renal Cancer | edgeR (Robinson *et al.*, 2010) | 72% | 100% (5/5) |

^1^Oasis uses a negative binomial distribution as basis for its statistical evaluation of the differential expression. A very similar approach is taken by the edgeR package that has been used in the Renal Cancer study. The AD dataset was analyzed using the non-parametric Wilcoxon-Mann-Whitney test and the Psoriasis data was analyzed using a Pearson’s chi-squared test.

^2^Overlap of differentially expressed miRNAs comparing Oasis’ results to published data. The percentage is calculated in reference to the shorter DE list.

^3^Overlap of differentially expressed miRNAs that have been validated independently in addition to the sRNA-seq experiment.

The following three sections will explain the comparisons in some more detail.

***AD data***


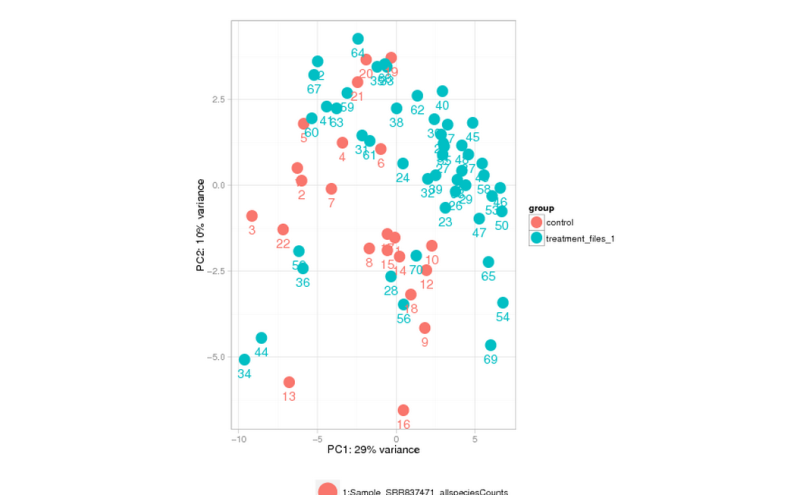
We started by analyzing the 48 AD and 22 control samples (Leidinger *et al.*, 2013) using Oasis’ default settings. By looking at the PCA plot (Supplementary Figure 2) it is possible to visualize a slight separation between AD and control.

Supplementary Figure 2. PCA plot comparing AD samples and the Normal control group.

The results of the original publication and Oasis show high overlap albeit using different statistical approaches to assess differential expression (Supplementary Table 4). The original work has found 125 annotated DE miRNAs and Oasis found 59 annotated DE miRNA (adjusted p-value <0.05) of which 40 overlapped with the original analysis (68%).

The original publication also contains a classification analysis used to discover a biomarker signature of AD. Unfortunately, we were unable to obtain the primary output of the SVM and could not follow the post-processing steps of the machine learning results as performed in the original publication (e.g. removal of miRNAs that also occur in other diseases). In brief, the original publication provides a biomarker signature of 12 miRNAs (10 annotated and two novel) that reaches an average accuracy of 80%. Oasis’ classification reaches an A.U.C. of 0.92 using all features and has an out-of-the-bag error of ~16% using the top 9 features (miRNAs) including 2 miRNAs in the original paper list (has-miR-151a-3p, has-miR-26a-5p) (Supplementary Figure 3 A, B and C).


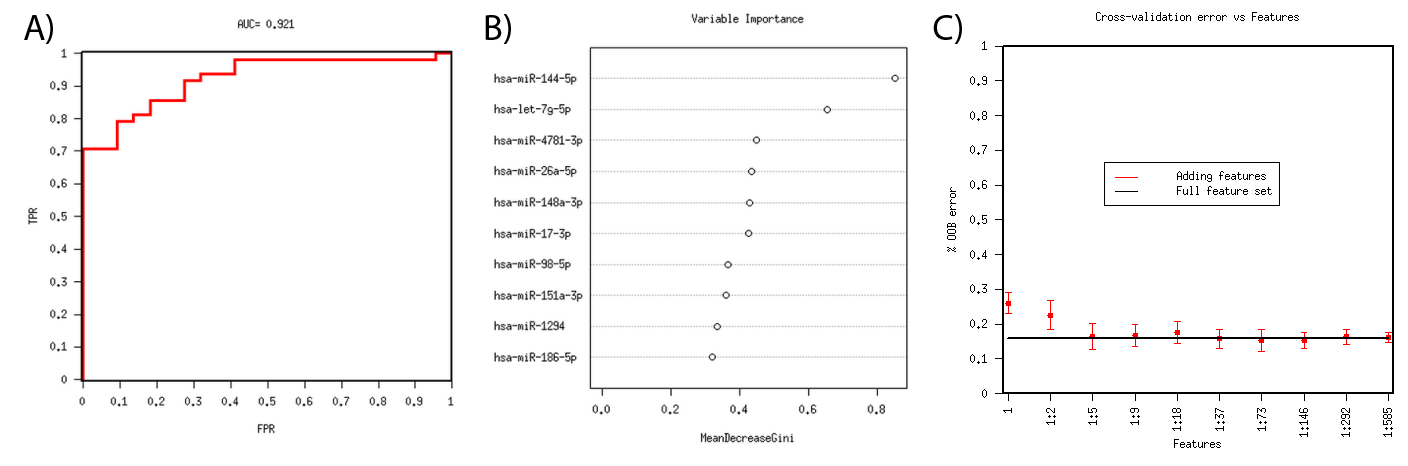


Supplementary Figure 3. Classification module quality and evaluation plots, including: A) ROC curve, B) Variable importance plot sorted by Gini index, C) Cross-validation prediction error by incrementally adding features.

As the authors included patient age and gender information for each sample, we ran Oasis again adding this covariate information (age is one of the top risk factors in AD) in order to obtain more accurate results. After this correction we obtained 66 significantly changed annotated miRNAs (and an additional 5 predicted miRNAs).

***Psoriasis data***

The second dataset we chose to validate Oasis’ biological performance on is a set of 10 Psoriasis cases and 10 healthy controls (see also demo datasets) (Joyce *et al.*, 2011). The original publication uses a hypergeometric test to assess differential expression (Pearson’s chi-square test) that is followed by Bonferroni multiple-testing correction. To make this comparison possible, we considered the pre-miRNA instead of the mature miRNA for both sides and the comparison is based on non-redundant pre-miRNAs. Oasis found 134 DE miRNAs whereas the original publication contains only 71 DE miRNAs. Of these 71 DE miRNAs 62% (44/71) could also be found in the significant Oasis miRNAs (Supplementary Table 4). Of note, the relatively poor overlap might in part be due to using a chi-squared test in the original publication, a non-optimal test for biological count data with replicate information.

Nonetheless, Oasis detected 75% (6/8) of all validated DE miRNAs (miR-21, miR-31, miR-124, miR-431, miR-933 and miR-3613) not detecting two validated miRNAs miR-124 and miR-219-2-3p that show high expression variation in the original publication.

Of note, Oasis’ PCA analysis highlights a potentially mis-annotated Psoriasis sample (Supplementary Figure 4, A) and another outlier sample (Supplementary Figure 4, B). Removal of these two samples increases the number of significantly DE miRNAs from 134 to 176 cases. We would like to emphasize that this data was already analyzed in two publications and to our knowledge this is the first time that these ‘problematic’ samples were detected, providing strong evidence for the utility of Oasis’ QC plots.

A

B


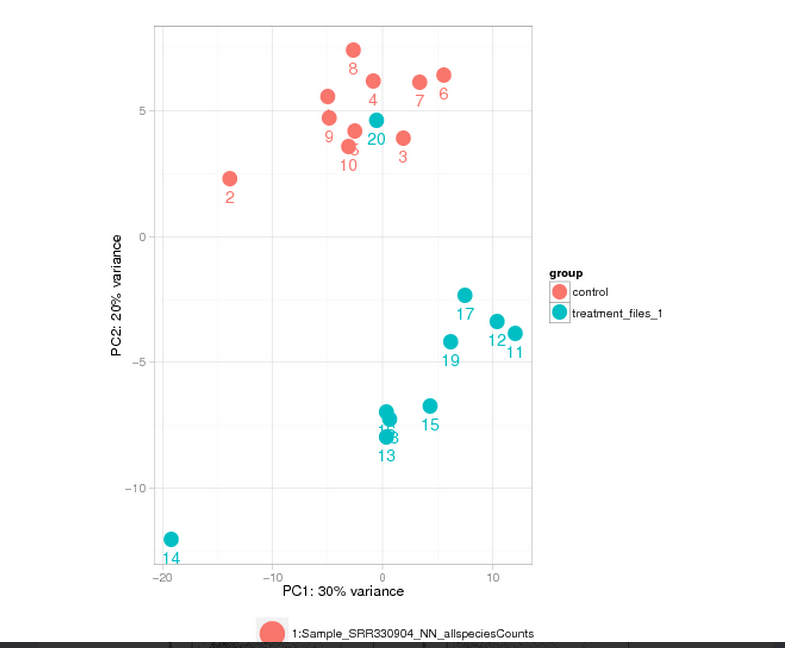


Supplementary Figure 4. PCA of psoriasis and control samples showing a misclassified (arrow A) and an outlier samples (arrow B).

***Renal cancer data***

Finally, we analyzed 11 Renal cancer and 11 remission samples (Osanto *et al.*, 2012). This is longitudinal data from 11 patients and as such paired. Unfortunately, we were not able to extract the pairing information from the GEO database annotations. Consequently we analyzed the data with Oasis in un-paired mode and compared it to the published, paired analysis of the authors using edgeR (Robinson *et al.*, 2010).

Despite of these technical problems the two analyses showed high overlap (72%) and all validated miRNAs were detected using Oasis (miR-21-5p, miR-122-5p, miR-210-3p, miR-199-5p, miR-532-5p).

In summary, our results provide firm evidence that Oasis provides biologically meaningful results to the user.

## Benchmark

In order to provide the user with runtime estimates, we have measured the execution time for every Oasis analysis module on the three provided demo datasets (AD, Psoriasis, and Renal cancer).

The compression of FASTQ files by OasisCompressor was measured on an Intel(R) Core(TM) i7-2600 processor with a clock Frequency of 3.4 GHz and 8GB of RAM. Every dataset was compressed using a single core making these estimates upper bounds. In brief, compression times ranged from 11 minutes for 1.9 GB of FASTQ files (22 Renal cancer files) to 5 hours 37,71 GB of FASTQ files (70 AD files) (Supplementary Table 5). All of the samples did not used less than 1 GB RAM at any time, providing easy and fast compression while keeping a low memory footprint.

Performance of the sRNA Detection, DE Analysis, and Classification modules were measured on the server. As a point of reference we compared Oasis’ performance to MAGI (Supplementary Table 5), taking all run-time estimates directly from MAGI’s website. Of note, we did not compare data upload times as the network latency varies for different servers.

**Supplementary Table 5.** Comparison of runtimes in the different Oasis modules.

| Demo Dataset | Oasis Compression | Oasis  sRNA Detection^1^ | Oasis  DE Analysis | Oasis Classification | MAGI  (total) |
| --- | --- | --- | --- | --- | --- |
| AD (287 GB)^4^ | 4h53m | 7h12m | 14m58s | 9m14s | NA^2^ |
| Psoriasis (48 GB) | 0h53m44s | 4h49m | 3m11s | 3m9s | 48h^3^ |
| Renal Cancer (9 GB) | 0h11m20s | 0h46m | 7m7s | 4m14s | 8h^3^ |

^1^ This running time includes the decompression of the data on server side

^2^ We could not get MAGI to upload all AD files. Most probably it has a problem with the quality or format of one of the files.

^3^ These values were obtained from the MAGI website.

## Supplementary references

Andrews,S. FastQC A Quality Control tool for High Throughput Sequence Data. *http://www.bioinformatics.babraham.ac.uk/projects/fastqc/*.

Betel,D. *et al.* (2008) The microRNA.org resource: Targets and expression. *Nucleic Acids Res.*, **36**.

Breiman,L. (2001) Random Forests. *Mach. Learn.*, **45**, 5–32.

Dobin,A. *et al.* (2013) STAR: ultrafast universal RNA-seq aligner. *Bioinformatics*, **29**, 15–21.

Fano,R.M. (1971) On the number of bits required to implement an associative memory. *Memo 61, Comput. Struct. Group, Proj. MAC, Massachusetts*.

Franceschini,A. *et al.* (2013) STRING v9.1: protein-protein interaction networks, with increased coverage and integration. *Nucleic Acids Res.* , **41** , D808–D815.

Friedländer,M.R. *et al.* (2012) MiRDeep2 accurately identifies known and hundreds of novel microRNA genes in seven animal clades. *Nucleic Acids Res.*, **40**, 37–52.

Friedman,R.C. *et al.* (2009) Most mammalian mRNAs are conserved targets of microRNAs. *Genome Res.*, **19**, 92–105.

Hsu,S. Da *et al.* (2014) MiRTarBase update 2014: An information resource for experimentally validated miRNA-target interactions. *Nucleic Acids Res.*, **42**.

Huang,D.W. *et al.* (2007) The DAVID Gene Functional Classification Tool: a novel biological module-centric algorithm to functionally analyze large gene lists. *Genome Biol.*, **8**, R183.

Joyce,C.E. *et al.* (2011) Deep sequencing of small RNAs from human skin reveals major alterations in the psoriasis miRNAome. *Hum. Mol. Genet.*, **20**, 4025–40.

Kuhn,M. *et al.* (2014) STITCH 4: Integration of protein-chemical interactions with user data. *Nucleic Acids Res.*, **42**.

Leidinger,P. *et al.* (2013) A blood based 12-miRNA signature of Alzheimer disease patients. *Genome Biol.*, **14**, R78.

Liao,Y. *et al.* (2014) featureCounts: an efficient general purpose program for assigning sequence reads to genomic features. *Bioinformatics*, **30**, 923–30.

Liaw,A. and Matthew,W. (2002) Classification and Regression by randomForest. *R News*, **2**, 18–22.

Love,M. *et al.* (2014) Moderated estimation of fold change and dispersion for RNA-Seq data with DESeq2. *bioRxiv*.

Martin,M. (2011) Cutadapt removes adapter sequences from high-throughput sequencing reads. *EMBnet.journal*, **17**, 10.

Osanto,S. *et al.* (2012) Genome-wide microRNA expression analysis of clear cell renal cell carcinoma by next generation deep sequencing. *PLoS One*, **7**.

Reimand,J. *et al.* (2011) G:Profiler - A web server for functional interpretation of gene lists (2011 update). *Nucleic Acids Res.*, **39**.

Robinson,M.D. *et al.* (2010) edgeR: a Bioconductor package for differential expression analysis of digital gene expression data. *Bioinformatics*, **26**, 139–40.

Sing,T. *et al.* (2005) ROCR: Visualizing classifier performance in R. *Bioinformatics*, **21**, 3940–3941.

Witkos,T.M. *et al.* (2011) Practical Aspects of microRNA Target Prediction. *Curr. Mol. Med.*, **11**, 93–109.

Xiao,F. *et al.* (2009) miRecords: An integrated resource for microRNA-target interactions. *Nucleic Acids Res.*, **37**.

Zuberi,K. *et al.* (2013) GeneMANIA prediction server 2013 update. *Nucleic Acids Res.*, **41**.
